# Antitumor Effects of Ononin by Modulation of Apoptosis in Non-Small-Cell Lung Cancer through Inhibiting PI3K/Akt/mTOR Pathway

**DOI:** 10.1155/2022/5122448

**Published:** 2022-12-27

**Authors:** Guowei Gong, Kumar Ganesan, Qingping Xiong, Yuzhong Zheng

**Affiliations:** ^1^Department of Bioengineering, Zunyi Medical University, Zhuhai Campus, Zhuhai, Guangdong 519041, China; ^2^Guangdong Provincial Key Laboratory of Functional Substances in Medicinal Edible Resources and Healthcare Products, Hanshan Normal University, Chaozhou, Guangdong 521041, China; ^3^School of Chinese Medicine, The Hong Kong University, Hong Kong SAR 999077, China; ^4^Jiangsu Key Laboratory of Regional Resource Exploitation and Medicinal Research, Huaiyin Institute of Technology, Huai'an, 223003 Jiangsu, China

## Abstract

Lung cancer is a leading global cause of cancer-related death in both males and females. Non-small-cell lung cancer (NSCLC) is the most commonly diagnosed cancer type that can be difficult to control with conventional chemotherapeutic and surgical approaches resulting in a poor prognosis. Paclitaxel (PTX) is a commonly used chemotherapeutic drug for NSCLC, which can cause tissue injury in healthy cells and affect the quality of life in patients with cancer. In order to treat NSCLC, alternative medications with minimal or no side effects are highly needed. Ononin is an isoflavone glycoside extracted from Astragali Radix (AR) that has various pharmacological activities. Therefore, this study investigated whether ononin inhibits NSCLC progression and promotes apoptosis synergistically with PTX both *in vitro* and *in vivo.* Antitumorigenic properties of ononin were determined by MTT assay, colony formation assay, migratory capacity, and apoptotic marker expression in A549 and HCC827 cells. The combination of ononin with PTX increased the expression of apoptotic markers and ROS generation and inhibited cell proliferation through the PI3K/Akt/mTOR signaling pathways. Furthermore, ononin prevented the translocation of NF-*κ*B from cytosol to the nucleus. Also, we used the xenograft NSCLC mice model to confirm the *in vivo* antitumorigenic efficacies of ononin by reduction of CD34 and Ki67 expressions. Based on the histological analysis, the cotreatment of PTX and ononin reduced PTX-induced liver and kidney damage. Overall, our findings suggested that the therapeutic index of PTX-based chemotherapy could be improved by reducing toxicity with increasing antitumor capabilities when combined with ononin.

## 1. Introduction

Lung cancer is the main cause of cancer-related mortality worldwide, with small-cell lung carcinoma (SCLC) and non-small-cell lung carcinoma (NSCLC) accounting for 80-85 and 15-20% of all lung cancer cases, respectively [1]. Despite technological advances, patients suffering from NSCLC continue to have limited life durations and a high mortality rate [2]. Conventional treatments of lung cancer, including paclitaxel- (PTX-) based chemotherapy and/or radiation therapy, are fairly ineffective and commonly result in liver and kidney dysfunction [3]. As a result, new alternative medications and less hazardous therapeutic techniques for the treatment of NSCLC are required. Medicinal plants have a long history of usage as an anticancer medicine source, and it has had a considerable impact on the modern drug development process [4]. In addition to improving the effectiveness of and reducing the side effects of chemotherapy, medicinal plants and their active principles have shown potential as new sources of anticancer drugs. Over the last 20 years, medicinal plants have provided more than 25% of drug candidates, while chemically modified natural products have provided another 25% [5].

Plant-derived anticancer medications include etoposide, which is derived from epipodophyllotoxins, topotecan and irinotecan, which are derived from camptothecins, and vincristine and vinblastine, which are produced from vinca alkaloids [6]. Similarly, flavonoids are also potentially promising compounds for the chemoprevention and chemotherapy of cancer. Several *in vitro* and *in vivo* studies demonstrated that flavonoids promote the inhibition of cell proliferation, cell differentiation, cell cycle phases, cell adhesion, invasion, and migration resulting in the inhibition of cancer progression [7–9]. This effect was chiefly characterized as being connected to molecular events during the stages of initiation, promotion, and development [10].

Ononin (formononetin-7-O-*β*-D-glucoside) is a class of flavonoids, primarily found in Astragali Radix, *Smilax scobinicaulis*, *Ononis angustissima*, *Millettia nitida*, soy beans, fruits, and other foods. It has numerous biological activities, including antioxidant, antidiabetic, antiobesity, neuroprotective, cardioprotective, antiviral, and anti-inflammatory properties [11–16]. A growing body of research has also revealed that ononin has antibreast cancer and antiprostate cancer activities in a dose- and time-dependent manner [10]. Furthermore, ononin prevented doxorubicin-induced cardiotoxicity by inhibiting ER stress and apoptosis, potentially stimulating the SIRT3 pathway [17]. PTX is a potent chemopreventive drug used for the treatment of a wide variety of cancer types. However, resistance among cancer cells has developed as a key barrier to effective clinical treatment with PTX resulting in death-associated treatment failure [18]. Studies showed that PTX is an antineoplastic drug for NSCLC, which significantly caused tissue injury in healthy cells and affected the quality of life in patients with cancer [19]. In order to treat NSCLC, alternative medications with minimal or no side effects are highly needed. Hence, the present study is aimed at investigating whether ononin inhibits NSCLC progression and promotes apoptosis synergistically with PTX in A549 and HCC827 cells and A549 xenograft mice. In this study, we sought to investigate the anti-NSCLC efficacy of ononin on A549 and HCC827 cells by examining (i) cell population, mitochondrial membrane potential (MMP) level, and reactive oxygen species (ROS) content; (ii) cell migration abilities and colony formation; (iii) cell apoptosis marker expression levels; (iv) A549 xenograft mice body and tumor weight; and (v) the transduction mechanism involved in both *in vitro* and *in vivo*.

## 2. Materials and Methods

### 2.1. Cell Culture

A549 and HCC827 were obtained from the American Type Culture Collection (ATCC, Manassas, VA) and cultured in Dulbecco's modified Eagles medium supplemented with 100 IU/mL penicillin, 100 g/mL streptomycin, and 10% fetal bovine serum. The firefly luciferase gene (luc2) has been inserted into the A549 cell line. Cells were cultured at 37°C in a water-saturated 5% CO2 incubator.

### 2.2. Animals

Shanghai Laboratory Animal Company supplied BALB/c nude mice (8-10 weeks) (Shanghai, China). The nude mice were kept in pathogen-free settings at 22 ± 2°C, 70% relative humidity, and a 12-hour light/dark cycle. A549 cells (5 × 10^9^/mL) were inserted subcutaneously into each mouse's flank. Tumors that were palpable and quantifiable were discovered 6 days following cell injection. The animals were then randomly assigned to one of six treatment groups: the vehicle control group received Milli-Q water; the PTX group received PTX (5 mg/kg/p.o.); the ononin-L, -M, and -H group received ononin (1 mg/kg/p.o., 3 mg/kg/p.o., and 10 mg/kg/p.o.); and the PTX combined ononin group received PTX (5 mg/kg/p.o. and 3 mg/kg/p.o.). All experimental procedures were authorized by the Zunyi Medical University's Animal Experimentation Ethics Committee (No. 21-015 for Animal Ethics Approval) and followed the “Principles of Laboratory Animal Care.” After 21 days of treatment, the mice were sacrificed, and the tumor weights were recorded. PTX was purchased from Sigma-Aldrich (T7402-25 mg).

### 2.3. MTT Assay

Cell viability was determined using the MTT test (Sigma-Aldrich, St. Louis, MO). Cells were grown in 96-well plates. After 48 hours of drug treatment, MTT solution was added to the cultures at a final concentration of 0.5 mg/mL. After 2 hours of incubation, the purple crystal growth was dissolved using DMSO. The absorbance was set at 570 *μ*M. Cell viability was calculated using the percentage of absorbance of the blank group, and the value was set to 1.

### 2.4. Flow Cytometry Analysis

Cells were cultured in 35 mm culture plates for 24 hours before being treated with medication. Flow cytometry was utilized for analyzing the cells using Annexin V-FITC/propidium iodide (PI) or JC-1 green/JC-1 red detection kits (BD Biosciences, Franklin Lakes, NJ). Before collecting cells in PBS, they were washed twice with phosphate-buffered saline (PBS). Cells were incubated for 15 minutes at room temperature in the dark in 100 *μ*L of binding buffer. The samples were automatically acquired using the loader with 10,000 event detection criteria for each tube, and the quadrants were constructed based on the viable population. The FlowJo v10.6 software was used to evaluate the results.

### 2.5. Production of ROS

An Olympus FV1000 laser scanning confocal system (Olympus America Inc., Melville, NY) installed on an inverted Olympus microscope with a 10x objective was used to detect ROS using the CellROX Green detection kit (BD Biosciences). Using the prescribed techniques, all measurements were revealed.

### 2.6. Migration Assay

In a sterile 12-well plate, 2 × 10^5^ cells were seeded in 1 mL of medium. By removing linked cells from the centre of the cell monolayer with a sterile 200 L plastic pipette tip, a single straight wound was produced. After 48 hours of drug therapy, cells' wound areas were randomly selected and snapped. The photos were captured using bright-field microscopy at a magnification of 10x (Olympus America Inc.).

### 2.7. Colony Formation Assay

1000 A549 or HCC827 cells were grown in a 6-well plate and treated with medication for 48 hours before being replaced with a fresh medium for another 7 days. The cells were fixed in methanol for 15 minutes at room temperature before being stained with crystal violet (Sigma-Aldrich) for 10 minutes.

### 2.8. Separation of Cytosolic and Nuclear Protein

After 48 hours of drug treatment, the cultures were washed with 1× PBS, followed by the addition of the Qproteome Nuclear Protein kit (QIAGEN, San Francisco, CA) to separate nuclear and cytosolic NF-*κ*B p65 protein. The cells were dissolved in lysis buffer (0.125 M Tris-HCl, pH 6.8, 4 percent SDS, 20% glycerol, and 2% 2-mercaptoethanol) for the western blot.

### 2.9. Western Blot

SDS-PAGE was used to determine the levels of protein expression of target genes in cell lysates. After transferring the target proteins to membranes, the membranes were incubated with anti-PARP (CST, Danvers, MA) at 1 : 1000 dilutions, anti-Cl-Caspase 9 (CST) at 1 : 2,000 dilutions, anti-Cl-Caspase 3 (CST) at 1 : 2,000 dilutions, anti-GAPDH (CST) at 1 : 5,000,000 dilutions, anti-P-PI3K (CST) at 1 : 5,000 dilutions, anti-T-PI3K (CST) at 1: 5,000 dilutions, anti-P-Akt (CST) at 1 : 5000 dilutions, anti-T-Akt (CST) at 1: 5,000 dilutions, anti-mTOR (CST) at 1: 5,000 dilutions, anti-p65 (CST) at 1 : 5,000, and antihistone-1 (CST) at 1: 5,000,000 dilutions at 4°C for overnight. The immunological complexes were detected by the enhanced chemiluminescence (ECL) method after 3 hours of incubation with horseradish peroxidase- (HRP-) conjugated secondary antibodies at room temperature (Amersham Biosciences, Piscataway, NJ). ImageJ was used to analyze the band intensities.

### 2.10. Translocation of NF-*κ*B p65 Assay

An Olympus FV1000 confocal system mounted on an inverted Olympus microscope was used to detect the p65 translocation (40x objective). After 10 minutes of fixation in 4 percent methanol-free paraformaldehyde, the p65 antibodies were diluted in cold PBS containing 2.5% fetal bovine serum and 0.1 percent Triton X-100 (Sigma-Aldrich). After overnight storage in a cold room, the samples were incubated in the dark for 3 hours at room temperature with a 1 : 1,000 dilution of FITC-labeled anti-rabbit antibody (Jackson Laboratories, West Grove, PA). The cells were washed three times after treatment, and the nucleus was stained with DAPI (1 : 5,000 dilution) before the experiment.

### 2.11. Bioluminescence Assay in the Xenograft Model

The mice were anesthetized with isoflurane and then intraperitoneally injected with 100 *μ*L of aminoluciferin (0.5 mM, diluted by normal saline), followed immediately by bioluminescence imaging for 3 minutes, and then, animals were sacrificed for further study. Photos were captured using the IVIS® Spectrum imaging system (PerkinElmer, Alameda, CA).

### 2.12. Histological Analysis

After 21 days of oral treatment, mice were sacrificed; tumors and organs were dissected, weighed, and photographed. Histological studies were performed using hematoxylin and eosin (H&E) staining. To measure the expressions of CD34, Ki67, P-PI3K, P-Akt, mTOR, and p65, immunohistochemical (IHC) analysis and western blot analysis were conducted.

### 2.13. Statistical Analyses and Other Tests

Protein concentrations were estimated using Bio-Bradford Rad's protein assay dye (Bio-Rad Laboratories, Hercules, CA) and BSA as standards. One-way ANOVA (Bonferroni's post hoc test) was used to assess the means of untreated control cells versus treated cells. Significant values were denoted by ^∗^*p* < 0.05, ^∗∗^*p* < 0.01, and ^∗∗∗^*p* < 0.001 when compared to the control group. When compared to the PTX group, significant values were indicated by ^*p* < 0.05, ^^*p* < 0.01, and ^^^*p* < 0.001.

## 3. Results

### 3.1. Ononin Exhibits Antiproliferative Activity against Human Lung Cancer A549 and HCC827 Cells

**Figure 1:**
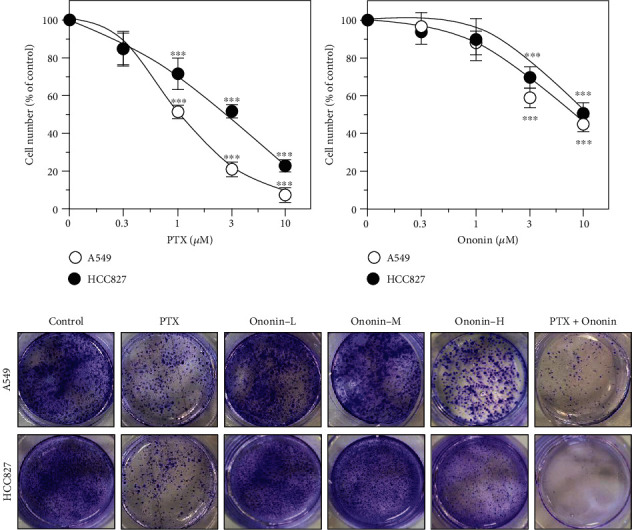
Ononin shows anticell proliferative effects. Cultured cells were treated with (a) PTX (0.3-10 *μ*M) or (b) ononin (0.3-10 *μ*M) for 48 hours. The cell viability was determined by MTT assay. Data are presented as a percentage change from the control group and in mean ± SEM, with *n* = 6. Statistical changes were clustered as significant ^∗∗∗^ where *p* < 0.001 as compared with the control group. (c) After 48 hours of drug treatment, cells were seeded in a 6-well plate and replaced with fresh DMEM for another 7 days. Crystal violet was used to stain the colony formation assay.

Firstly, we optimized the ideal concentration of PTX on A549 or HCC827 cells before proceeding with the biological analysis. We used the MTT test to detect the optimal concentration of PTX on two cell types (Figure 1(a)). PTX suppressed cell proliferation of A549 or HCC827 cells in a dose-dependent manner. PTX at 1 *μ*M was found to be minimally toxic to HCC827 or A549 cells in comparison to the untreated group. As a result, PTX at 1 *μ*M was selected for the following experiments (Figure 1(a)). On the other hand, we used the MTT assay to determine the different concentrations of ononin in A549 or HCC827 cells (Figure 1(b)). The results showed that ononin at 3 *μ*M induces cell death in both A549 and HCC827 (Figure 1(b)). Furthermore, we performed a colony formation assay to verify the cotherapy effect of ononin and PTX for NSCLC treatment (Figure 1(c)). Based on preliminary data, cotreatment of ononin with PTX reduced colony formation *in vitro* as compared to PTX used alone (Figure 1(c)).

### 3.2. Ononin Promotes Apoptosis and Decreases Mitochondrial Membrane Potential in Human Lung Cancer A549 and HCC827 Cells

**Figure 2:**
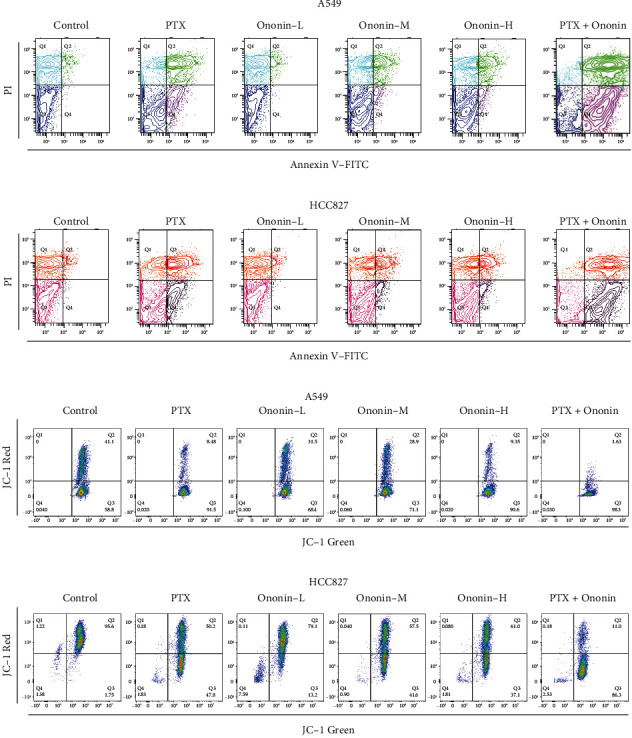
Ononin stimulates NSCLC apoptotic ability. (a, b) The viable cell population was displayed in the bottom left quadrant (Q3), the early apoptotic cells in the bottom right quadrant (Q4), and the late apoptotic cells in the top right quadrant (Q4) of the dual parametric dot plots integrating Annexin V-FITC and PI fluorescence (Q2). (c, d) The MMP level was detected by a flow cytometry with fluorescent dye JC-1. Cytogram plots of control and samples incubated with PTX (1 *μ*M, positive control) or ononin (0.3, 1, and 3 *μ*M) or the combination of PTX (1 *μ*M) and ononin (1 *μ*M) for 48 h, respectively. The horizontal axis showed the green fluorescence (JC-1 monomer), and the vertical axis indicated red fluorescence (JC-1 aggregates).

The induction of cell apoptosis was studied in A549 or HCC827 cells treated with 1 *μ*M PTX or different concentrations of ononin (Figures 2(a) and 2(b)). Flow cytometry was used to examine Annexin V-FITC- and PI-labeled A549 or HCC827 cells, which exhibited apoptosis after PTX therapy. PTX at 1 *μ*M showed a significant induced apoptotic rate as compared to the control group on A549 or HCC827 cells (Figures 2(a) and 2(b)). Furthermore, a succession of ononin treatments boosted the rate of cell death as reflected by a flow cytometer. More importantly, cotreatment of ononin with PTX increased the rate of cell death significantly when compared to PTX alone (Figures 2(a) and 2(b)).

**Figure 3:**
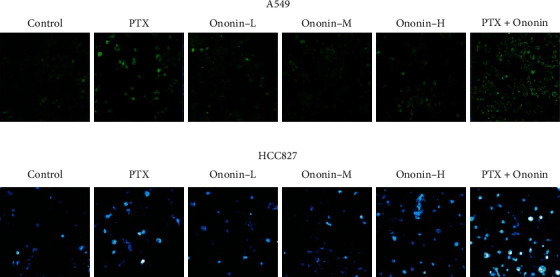
Ononin enhances ROS production in NSCLC. Cultured cells were treated with PTX (1 *μ*M) or ononin (0.3, 1, and 3 *μ*M) or the combination of PTX (1 *μ*M) and ononin (1 *μ*M) for 48 h, respectively. The ROS formation was evaluated by measuring the fluorescence intensities.

Mitochondrial membrane potential (MMP) is an incredibly crucial aspect of mitochondrial health since it is required to sustain the electrochemical potential of hydrogen ions required to synthesize ATP via the electron transport chain [20, 21]. MMP is reasonably constant throughout the typical physiological activity. Long-term disruption, on the other hand, has a negative impact on cellular health and viability, leading to a variety of pathophysiologies such as diabetes, cancer, and neurodegeneration [16]. MMP level was found after 48 hours in the presence of ononin or PTX using a JC-1-based detection kit and flow cytometry analysis (Figures 2(c) and 2(d)). When MMP levels were altered in A549 or HCC827 cells, there was an increase in cell death caused by ononin or PTX when compared to the control group. Cotreatment of PTX with ononin significantly reduced MMP levels when compared to the PTX-treated group alone (Figures 2(c) and 2(d)). Also, we used confocal microscopy to detect ROS production in the presence of PTX or ononin (Figures 3(a) and 3(b)). Results showed that ROS production was increased in a dose-dependent manner by ononin on A549 or HCC827 cells (Figures 3(a) and 3(b)). The cotreatment group showed the greatest effectiveness in terms of ROS formation in comparison to the single therapy (Figure 3).

**Figure 4:**
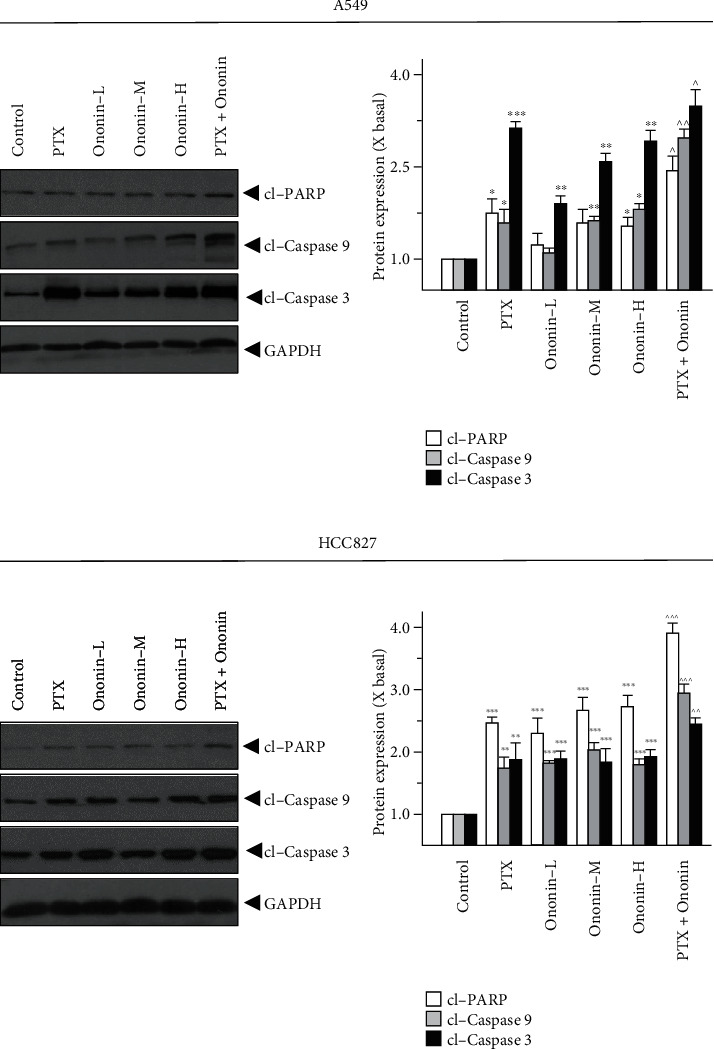
Ononin initiates apoptosis biomarker expressions. Cultured A549 or HCC827 cells were incubated with PTX (1 *μ*M) or ononin (0.3, 1, and 3 *μ*M) or the combination of PTX (1 *μ*M) and ononin (1 *μ*M) for 2 days, and the target proteins were detected by western blot. The quantification of the target protein was calculated by a densitometer. The values are expressed as the fold of changes (X basal), in mean ± SEM, where *n* = 6. ^∗^*p* < 0.05, ^∗∗^*p* < 0.01, and ^∗∗∗^*p* < 0.001 when compared to the control group. When compared to the PTX group, significant values were indicated by ^*p* < 0.05, ^^*p* < 0.01, and ^^^*p* < 0.001.

Ononin treatment for 48 hours dramatically increased cell apoptosis by increasing the expression of apoptosis biomarkers such as cleaved-PARP, cleaved-caspase 3, and cleaved-caspase 9 on A549 or HCC827 cells (Figure 4). Ononin triggered NSCLC cell death in a concentration-dependent manner by revealing translational activity of cleaved-PARP, cleaved-caspase 3, and cleaved-caspase 9. The combination of PTX and ononin significantly elevated the levels of these apoptotic markers (Figure 4). The cotreatment increased cleaved-PARP by 2.5-fold, cleaved-caspase 3 by 3.2-fold, and cleaved-caspase 9 by 2.8-fold in A549 cells when compared to the control group. Likewise, the cotreatment of PTX and ononin stimulated cleaved-PARP by 3.8-fold, cleaved-caspase 3 by 2.5-fold, and cleaved-caspase 9 by threefold on HCC827 cells, respectively (Figure 4).

### 3.3. Ononin Blocks PI3K/Akt/mTOR Signaling in Human Lung Cancer A549 and HCC827 Cells

**Figure 5:**
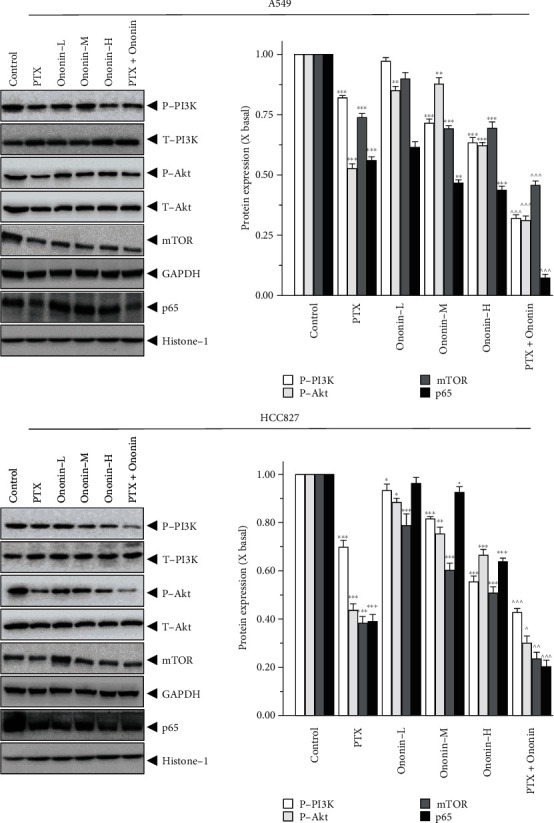
Ononin abolishes PI3K/Akt/mTOR expression levels. Cultured cells were incubated with PTX (1 *μ*M) or a series concentration of ononin (0.3, 1, and 3 *μ*M) or the combination of PTX and ononin for 2 days, and the target proteins were detected by western blot. The quantification of the target protein was calculated by a densitometer. The values are expressed as the fold of changes (X basal), in mean ± SEM, where *n* = 6. ^∗^*p* < 0.05, ^∗∗^*p* < 0.01, and ^∗∗∗^*p* < 0.001 when compared to the control group. When compared to the PTX group, significant values were indicated by ^*p* < 0.05, ^^*p* < 0.01, and ^^^*p* < 0.001.

**Figure 6:**
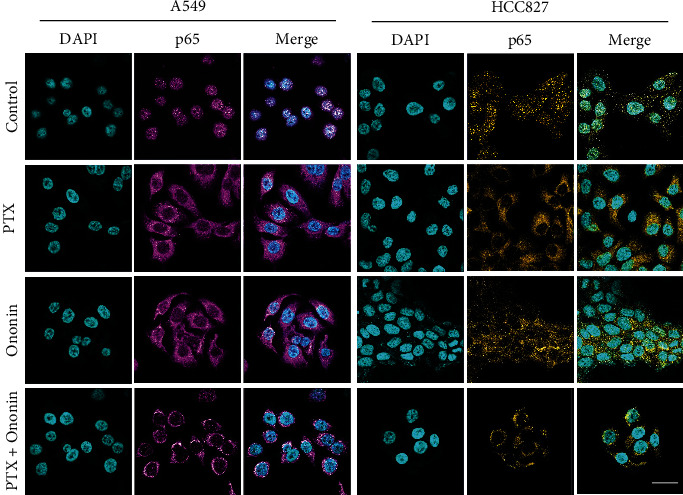
Ononin restricts p65 translocation. p65 expression in cultured cells was observed by laser confocal microscope. After drug treatment for 48 hours, the amount of p65 (shown in purple) was revealed by confocal microscopy. Nuclei were stained by DAPI (shown in blue color). Bar = 20 *μ*M.

To investigate the underlying molecular mechanism of ononin-induced apoptosis, the levels of proteins involved with the PI3K/Akt/mTOR signaling pathway were measured by western blotting (Figure 5). The findings indicated that, when compared to the control group, P-PI3K, P-Akt, mTOR, and NF-*κ*B isolated from the nucleus were dramatically downregulated in A549 or HCC827 cells after 48 hours of treatment with varying concentrations (0.3, 1, and 3 *μ*M) of ononin (Figure 5). In addition, we used the specific PI3K/Akt agonist, 740Y-P (Selleckchem, Houston, TX) to further validate the transduction of ononin for treating NSCLC *in vitro* (Supplementary Figure 1). From the western blot data, we found that 740Y-P promoted PI3K/Akt activation. After cotreatment of ononin with 740Y-P, the translational level of PI3K/Akt decreased as compared to the agonist group (Supplementary Figure 1). Furthermore, we used a laser confocal microscope to assess the translocation of NF-*κ*B p65 after PTX or ononin therapy in A549 or HCC827 cells (Figure 6). The results showed that the translocation of p65 was reduced by PTX or ononin therapy as compared to the control group.

### 3.4. Ononin Inhibits Migratory Activity in Human Lung Cancer A549 and HCC827 Cells

**Figure 7:**
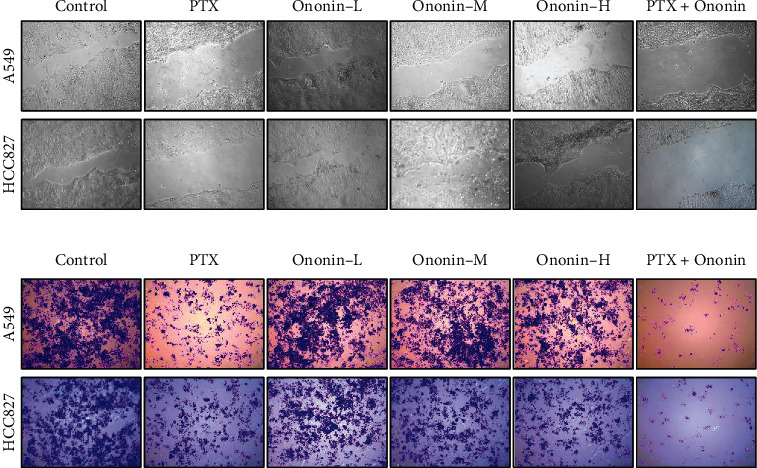
Ononin suppresses cell migration. (a) Wound-healing and (b) transwell assays were utilized to determine antimigration effect of ononin after drug treatment for 48 hours.

**Figure 8:**
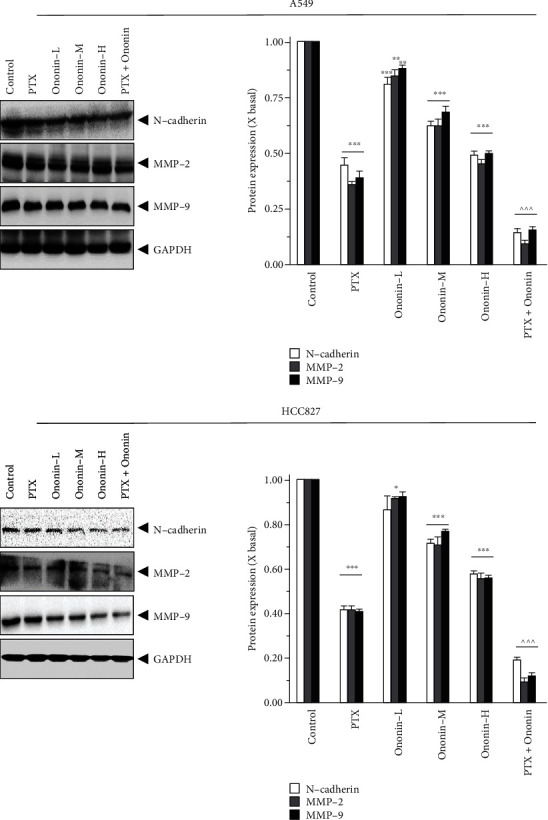
Ononin decreases metastasis marker translational levels. Cultured cells were incubated with PTX (1 *μ*M) or a series concentration of ononin (0.3, 1, and 3 *μ*M) or the combination of PTX and ononin for 2 days, and the target proteins were detected by western blot. The quantification of the target protein was calculated by a densitometer. The values are expressed as the fold of changes (X basal), in mean ± SEM, where *n* = 6. ^∗^*p* < 0.05, ^∗∗^*p* < 0.01, and ^∗∗∗^*p* < 0.001 when compared to the control group. When compared to the PTX group, significant values were indicated by ^^^*p* < 0.001.

To confirm the migration activity of A549 or HCC827, we used wound-healing assays and transwells (Figure 7). After 48 hours of treatment, we found that PTX could limit migration effect at 1 *μ*M, and ononin could also inhibit migration function, dose-dependently. However, in terms of inhibiting cell migration, the combination of PTX and ononin demonstrated a superior therapeutic effect when compared to the PTX alone group on A549 or HCC827 cells (Figure 7(a)). Furthermore, we found that ononin treatment significantly reduced cell migratory functions by using transwell (Figure 7(b)). More importantly, we also employed SDS-PAGE to determine the protein expressional levels of biomarkers during the migration process (Figure 8). From the preliminary data, we found ononin inhibited the N-cadherin, MMP-2, and MMP-9 dose-dependently on A549 and HCC827 cells (Figure 8). The cotreatment group showed the strongest anticell migration effect when compared to PTX or ononin treatment alone (Figure 8).

### 3.5. Ononin Inhibits Lung Tumor Growth in A549 Xenograft Mice Models

**Figure 9:**
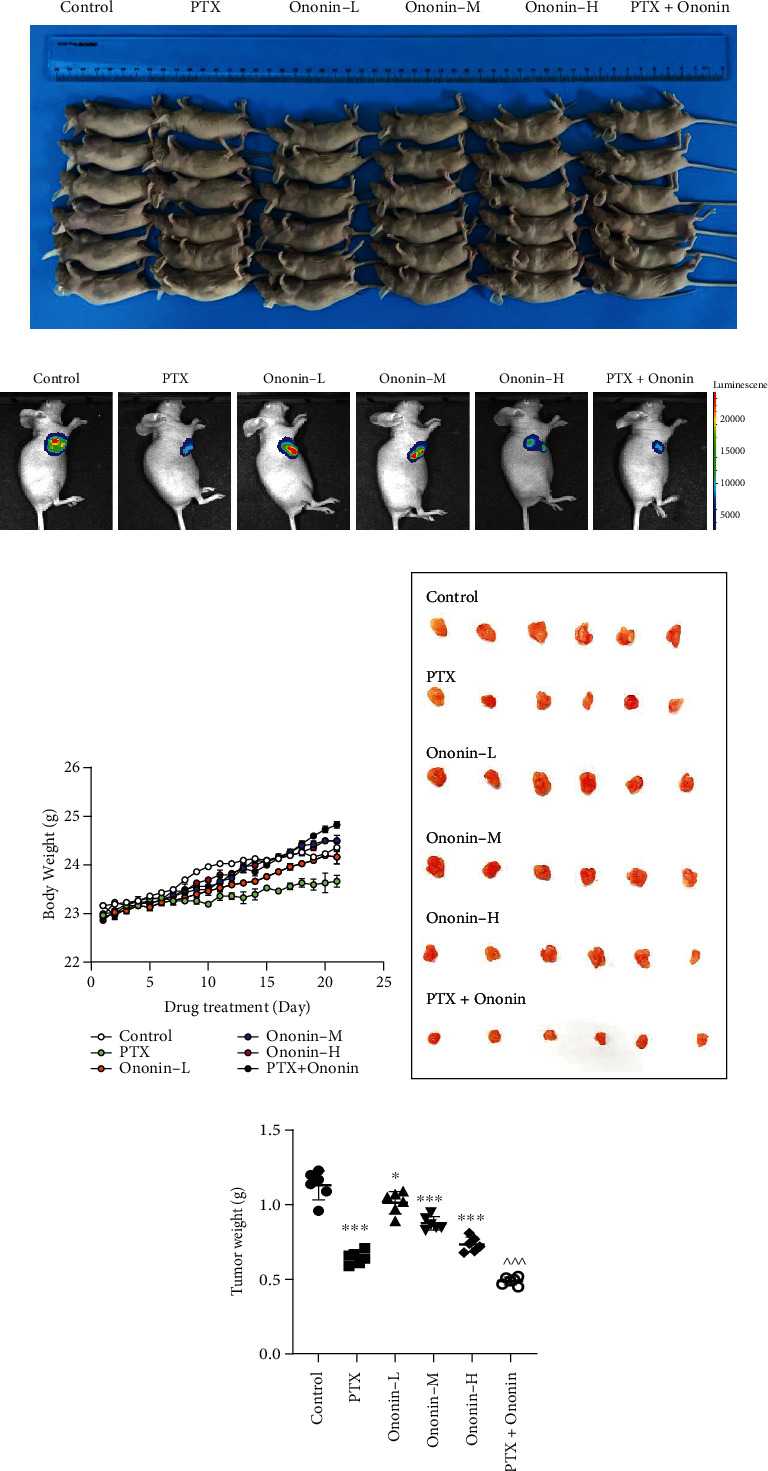
Ononin shows anti-NSCLC effect on A549 xenograft mice. (a) Picture of A549 xenograft nude mice after drug treatment for 21 days. (b) A bioluminescence imaging of A549-luc xenograft mice after drug treatment for 21 days, showing the preventive effects of PTX, ononin-L, ononin-M, ononin-H, and PTX with ononin on NSCLC development of A549-luc xenograft mice. Photos were captured using the IVIS® Spectrum imaging system, and the fluorescence was analyzed by the Living Image software. (c) The mice's body weight was recorded during the course of the 21-day investigation. (d) On day 21, tumors were removed from mice. (e) At the conclusion of the studies, the tumor weight was calculated. The data were reported as mean ± SEM, with *n* = 6. ^∗^*p* < 0.05 and ^∗∗∗^*p* < 0.001 when compared to the control group. When compared to the PTX group, significant values were indicated by ^^^*p* < 0.001.

Based on the promising results from the preliminary *in vitro* evidence, we used A549 xenograft mice models to validate the induction of apoptotic mechanisms by ononin (Figure 9). The tumor size was significantly decreased after 21 days of ononin and PTX treatment group compared to the control group (Figures 9(a) and 9(b)). Notably, the cotreatment of ononin with PTX dramatically reduced tumor size when compared with treatment with PTX alone (Figure 9(b)). The body weight decreased in the PTX chemotherapeutic group as compared with the control group (Figure 9(c)). The isolated tumor tissue and weight were markedly reduced in the PTX-treated group (Figures 9(d) and 9(e)). By contrast, in the ononin-treated group, tumor size and weight were lowered at a dose-dependent rate. Ononin was more effective when given at 10 mg/kg/p.o. (Figure 9). The cotreatment group demonstrated the most potent antitumor growth benefits when compared to single therapy groups (Figure 9).

**Figure 10:**
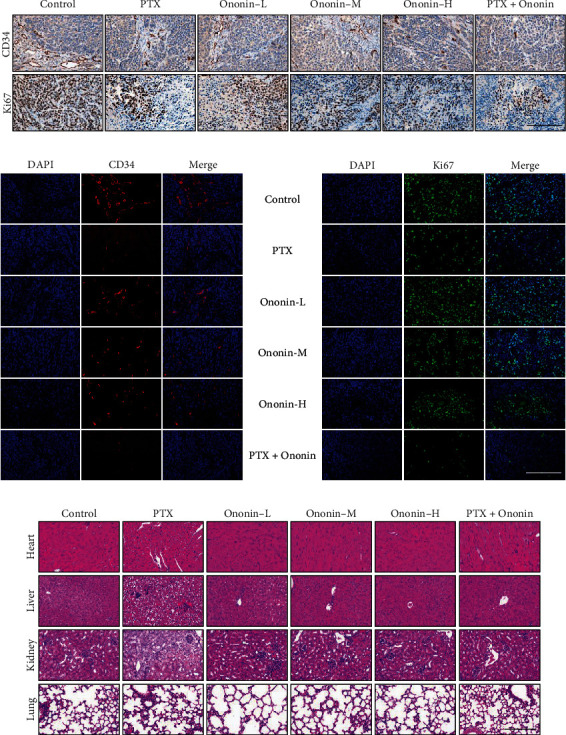
Ononin exhibits antiproliferation *in vivo*. The IHC staining (a) and laser confocal (b) method was utilized to verify the protein expression level of CD34 and Ki67. (c) HE staining of the heart, liver, kidney, and lung in the A549 xenograft mice obtained from different groups is shown here.

Histology and confocal microscopy were used to examine the protein expression of CD34 and Ki67 in tumor tissue of different treatment groups (Figures 10(a) and 10(b)). The CD34 and Ki67 levels were lowered in the PTX group in comparison to the control group. Similarly, CD34 and Ki67 levels were dose-dependently reduced in the ononin-treated group. Notably, cotreatment with PTX and ononin significantly reduced Ki67 and CD34 when compared to other groups (Figures 10(a) and 10(b)). To examine the toxicity study of ononin, we used different organs and tissues with HE (heart, liver, spleen, lung, and kidney), as shown in Figure 10(c). According to the findings, PTX is cytotoxic to the liver and kidney. However, there were no significant differences among the ononin or cotreated groups, which indicates that ononin is a safe molecule (Figure 10(c)).

### 3.6. Ononin Blocks PI3K/Akt/mTOR and Metastasis in A549 Xenograft Mice

**Figure 11:**
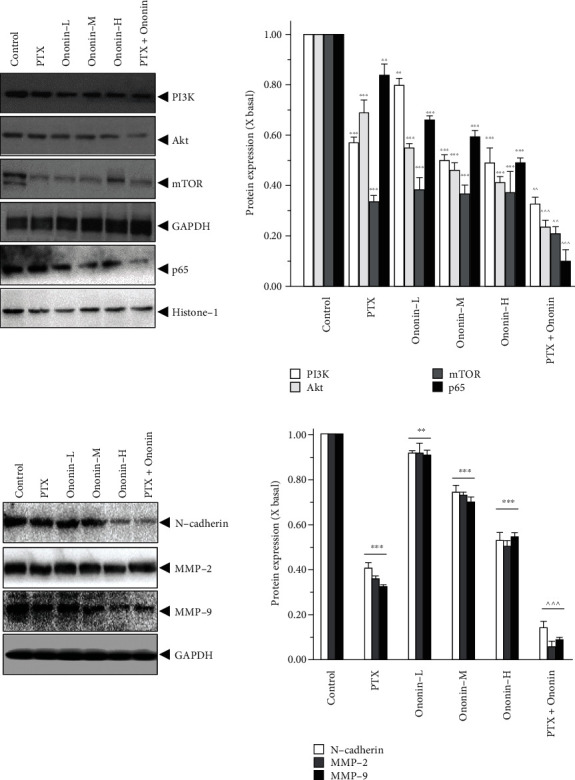
Ononin suppresses PI3K/Akt/mTOR and metastasis marker expression on A549 xenograft mice. (a, b) The homogenized tumor tissue was subjected to SDS-PAGE for analyzing the target protein expression levels. The quantification of the target protein was calculated by a densitometer. The values are expressed as the fold of changes (X basal), in mean ± SEM, where *n* = 6. ^∗∗^*p* < 0.01 and ^∗∗∗^*p* < 0.001 when compared to the control group. When compared to the PTX group, significant values were indicated by ^*p* < 0.05, ^^*p* < 0.01, and ^^^*p* < 0.001.

PI3K protein expression was also identified in tumor tissue (Figure 11(a)). In the cotreatment group, PI3K protein expression was lowered than in the PTX- or ononin-treated groups. Furthermore, this cotreatment therapy effectively blocked Akt and mTOR expressions (Figure 11(a)). We also isolated the p65 fraction from the nucleus, which decreased after being treated with a series of concentrations of ononin or PTX. The protein expression of p65 was lowered in the cotreatment groups than in the other group, indicating that the combination of PTX and ononin was more effective in suppressing NSCLC (Figure 11(a)).  Figure 11(b) shows the effects of PTX or ononin on metastasis biomarkers in the tumor tissues. In the cotreatment drug group, the expression levels of N-cadherin and MMP-2 and -9 were noticeably decreased as compared to the single drug treatment (Figure 11(b)). Overall, the results indicated that ononin can rejuvenate the chemotherapeutic effects of PTX while decreasing its toxicity.

## 4. Discussion

PTX has been recognized as a chemotherapeutic agent with clinically noteworthy efficacy against a variety of cancer types and has become the first-line therapy for NSCLC [22, 23]. On the other hand, prolonged administration and optimum doses of this drug can lead to serious side effects, such as neutropenia, neuropathy, and cancer cell resistance [24]. Nowadays, much attention has been given to discovering new therapeutic agents with synergistic effects with PTX. Accumulating evidence suggests that combination therapies have been shown to be more potent than monotherapy treatment in cancers [25–27]. They not only potentiate the treatment efficacy of each drug alone and/or allow the use of reduced doses of a single agent but also reduce the prospect of drug resistance [28, 29]. A shred of evidence suggests that the combination of plant-derived active compounds and chemotherapeutic drugs can have significant therapeutic benefits. Generally, plant-derived compounds have increased efficiency and reduced toxicity and can improve immunity by regulating many signaling pathways [30].

Astragali Radix (AR) is a vital tonic plant that possesses various pharmacological activities including immunomodulatory, cardioprotective, hepatoprotective, antioxidant, and anticancer properties [14, 31–33]. AR has a positive effect on various cancers including hepatocarcinogenesis and colon, lung, and gastric carcinoma [31, 34–36]. AR has been recognized as the antineoplastic agent that improves therapeutic outcomes in reducing toxicity during radiotherapy or chemotherapy. Similarly, a clinical trial also suggested that AR could improve the anticancer efficacy of PTX-based chemotherapy in NSCLC patients [36]. The extensive role and the related synergistic mechanisms of AR with cisplatin on laryngeal squamous cell carcinoma have also been well established [37]. Ononin is an active principle primarily found in AR that possesses various pharmacological properties including anticancer activities [38]. To date, there is no study related to the synergistic effects of ononin and PTX. Thus, this study sought to investigate whether ononin inhibits NSCLC progression and promotes apoptosis synergistically with PTX in A549 and HCC827 cells and A549 xenograft mice.

Inhibiting the proliferation of tumor cells is a key approach to treating cancer [39]. Thus, we examined the antiproliferative effect of ononin on human lung cancer cells, A549 and HCC827, by using both MTT and colony formation assays. The results showed that dose-dependent manners of ononin synergistically with or without PTX significantly inhibited the proliferation of A549 and HCC827 cells. Furthermore, the flow cytometry analysis reported that PTX at 1 *μ*M expressively increased the apoptotic rate; however, cotreatment with ononin significantly increased the rate of cell death and ROS generation and decreased MMP in human lung cancer A549 and HCC827 cells. This study was consistent with an earlier report that fisetin, a polyphenol, acted synergistically with PTX to induce the apoptosis and autophagy of A549 cells [24]. The production of ROS can also promote antitumorigenic effects, initiating oxidative stress-induced tumor cell death [40].

Cotreatment of dose-dependent ononin with PTX significantly increased the rate of apoptosis by increasing the expression of apoptosis markers, cleaved-PARP, cleaved-caspase 3, and cleaved-caspase 9 resulting in NSCLC cell death. Apoptosis is a type of programmed cell death that involves the degradation of cytoskeletal components, which is caspase-dependent [41, 42]. Thus, we detect whether ononin was able to induce apoptosis using biomarkers in A549 and HCC827 cells. Results indicated that ononin synergistically with PTX caused apoptotic induction of A549 and HCC827 cells. There is mounting evidence that PI3K/Akt/mTOR signaling influences key tumor progressions, including proliferation, migration, metastasis, and angiogenesis, by regulating downstream pathways [43, 44]. It is well recognized that aberrant changes in this signal are related to the development of NSCLC. Thus, we examined the expression levels of PI3K/Akt/mTOR in ononin or PTX or combined treated cells. The data reported that cotreatment of ononin with PTX or alone blocked the PI3K/Akt/mTOR signaling pathway in NSCLC. In both *in vitro* and A549-induced xenograft mice, results were consistent that the combined treatment of ononin with PTX blocked the PI3K/Akt/mTOR signaling pathway. This present study was consistent with the reports from earlier studies that treatment with salvianolic acid A, lutein, and betalain inhibited the PI3K/Akt/mTOR signaling pathway resulting in the inhibition of NSCLC [43, 45, 46]. The results of the present study demonstrated that ononin inhibited the translocation of p65 and thus inactivation of the NF-*κ*B signaling pathway. Through this inhibition, ononin and PTX are accumulated within tumor cells, increasing the toxic effects on cancer cells that result in NSCLC apoptosis. There were no significant differences among the ononin or cotreated groups, which indicates that ononin is a safe drug.

Our study is the first to show that ononin has anticancer activity against NSCLC by inducing cell apoptosis. This comparable anticancer impact was shown with the chemotherapeutic agent PTX in the xenograft model, and the outcome shows no evident cytotoxicity. As a result, ononin is offered as a viable drug for the development of innovative anticancer drugs for NSCLC.

## 5. Conclusion

PTX is an antineoplastic drug for NSCLC which may cause resistance among cancer cells that is a key barrier to effective clinical treatment. Ononin inhibits NSCLC progression synergistically with PTX in A549 and HCC827 cells and A549 xenograft mice. Specifically, cotreatment of ononin with PTX induced apoptosis, inhibiting cell proliferation, invasion, migration, and metastasis via blocking the PI3K/Akt/mTOR pathway which was further validated using biomarkers in human lung cancer A549 xenograft mice. Overall, our findings suggested that the therapeutic index of PTX-based chemotherapy could be improved by reducing toxicity with increasing antitumor capabilities when combined with ononin.

## Data Availability

The data used and/or analyzed during the current study are available from the corresponding author on reasonable request.

## Supplementary Materials

Supplementary Materials Supplementary Figure 1: ononin reverses 740Y-P activation. Cultured cells were incubated with 740Y-P (10 *μ*M) or combined with ononin (1 *μ*M) for 2 days, and the target proteins were detected by western blot. The values are expressed as the fold of changes (X basal), in mean ± SEM, where *n* = 3. ^∗∗^*p* < 0.01 and ^∗∗∗^*p* < 0.001 when compared to the control group. When compared to the 740Y-P group, significant values were indicated by ^*p* < 0.05 and ^^*p* < 0.01.
