# Supplementary figures and images for: Antitumor Effects of Ononin by Modulation of Apoptosis in Non-Small-Cell Lung Cancer through Inhibiting PI3K/Akt/mTOR Pathway
Source: Oxid Med Cell Longev. 2022 Dec 27;2022:5122448. doi: 10.1155/2022/5122448 (PMC9810408; doi:10.1155/2022/5122448)

**A**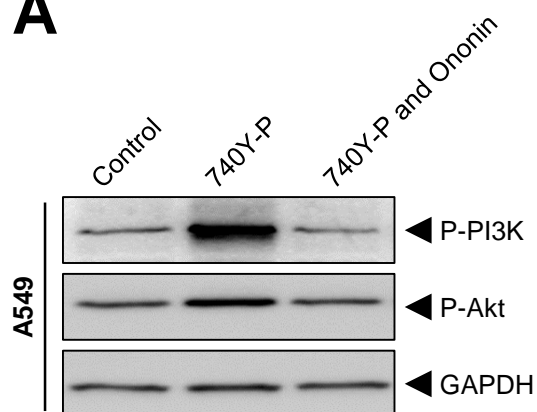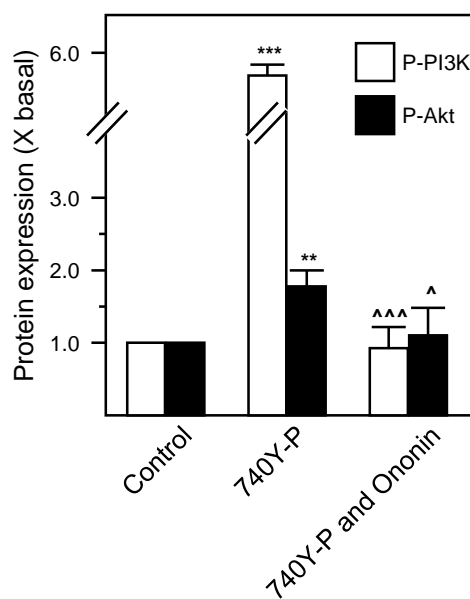**B**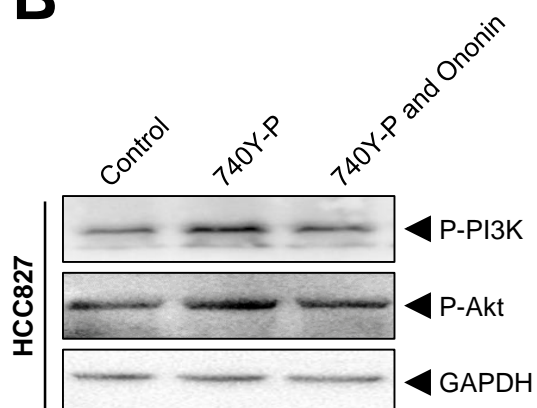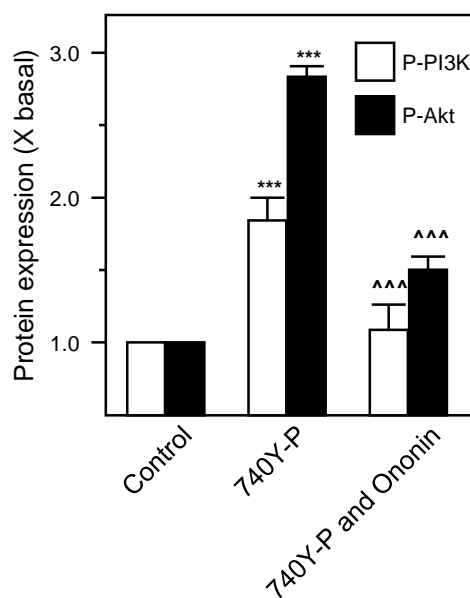

Supplement: Supplementary Materials — Supplementary Figure 1: ononin reverses 740Y-P activation. Cultured cells were incubated with 740Y-P (10 μM) or combined with ononin (1 μM) for 2 days, and the target proteins were detected by western blot. The values are expressed as the fold of changes (X basal), in mean ± SEM, where n = 3. ∗∗p < 0.01 and ∗∗∗p < 0.001 when compared to the control group. When compared to the 740Y-P group, significant values were indicated by ^p < 0.05 and ^^p < 0.01. [file 5122448.f1.pdf]
